# Global, regional, and national burden of hepatoblastoma, 1990–2021: a systematic analysis of the global burden of disease study 2021

**DOI:** 10.1097/JS9.0000000000002255

**Published:** 2025-01-29

**Authors:** Lang Yang, Zi-Qing Yu, Yi-Zhou Zhang, Yi-Qiao Deng, Xiao Chen, Hua-Yuan Liu, Xiao-Yin Bai, Hong Zhao

**Affiliations:** a Department of Hepatobiliary Surgery, National Cancer Center/National Clinical Research Center for Cancer/Cancer Hospital, Chinese Academy of Medical Sciences and Peking Union Medical College, Beijing, China; b Department of Gastroenterology, Peking Union Medical College Hospital, Chinese Academy of Medical Sciences & Peking Union Medical College, Beijing, China

*Dear Editor,*

Malignant liver tumors are exceedingly rare in children, comprising approximately 1–2% of all childhood malignancies[1]. However, among liver malignancies, hepatoblastoma constitutes more than 90% of hepatic malignancies in children under 10 years of age, with patients in this age group comprising over 95% of the total hepatoblastoma population[2]. In contrast to hepatocellular carcinoma, hepatoblastoma generally has a favorable prognosis[3], and can be effectively managed with chemotherapy, liver resection, and transplantation[4]. In the SIOPEL-4 trial, the 3-year overall survival rate was 83%, while in the AHEP0731 trial, the 5-year event-free survival rate was 88%[5]. Nonetheless, recent studies indicated an increasing trend in hepatoblastoma incidence[6]. Herein we utilized the latest GBD 2021 estimates to evaluate epidemiological trends in childhood hepatoblastoma from 1990 to 2021.

In GBD 2021, all liver cancer deaths occurring in individuals younger than 10 years old were classified as hepatoblastoma, which was ultimately integrated into the Causes of Death database to generate burden estimates. We obtained the age-standardized rate (ASR) of prevalence, incidence, mortality, and Disability-adjusted life-years (DALYs). To track burden trends, we used the generalized linear regression model to calculate estimated annual percentage change (EAPC) values of age-standardized prevalence rate (ASPR), age-standardized incidence rate (ASIR), age-standardized death rate (ASDR), and age-standardized DALYs. We also analyzed the relationship between socio-economic development, as measured by the Sociodemographic Index (SDI) and Human Development Index (HDI), and prevalence, incidence, mortality, and DALYs rate across countries and regions. Higher values of SDI and HDI indicate better socio-economic development.

## Global level

**Figure 1.**
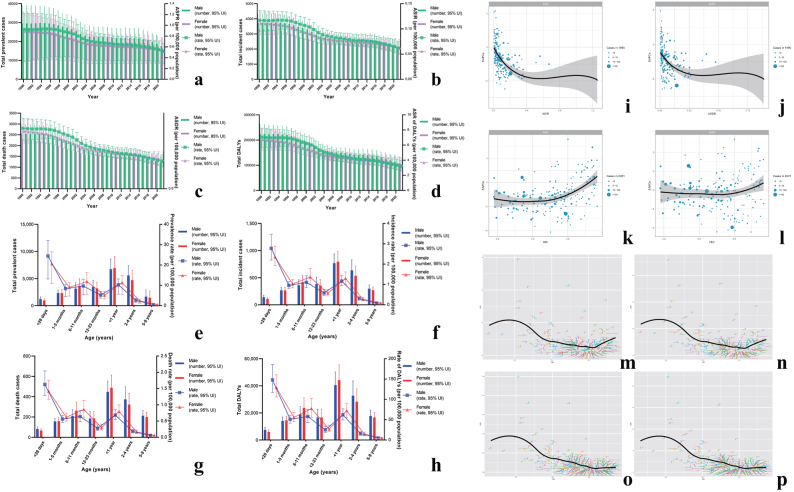
**(a**–**d):** Trends in hepatoblastoma burden worldwide, 1990-2021. The results indicated a downward trend in the burden of hepatoblastoma by both sexes during 1990-2021. (a) worldwide trends in total prevalent cases; (b) worldwide trends in total incident cases; (c) worldwide trends in total death cases; (d) worldwide trends in total DALYs. ASPR, age-standardized prevalence rate; ASIR, age-standardized incidence rate; ASDR, age-standardized death rate; DALYs, Disability-Adjusted Life Years. (e–h): Age and sex distribution of hepatoblastoma disease burden worldwide, 2021. The results indicated that for both males and females, the burden of disease was highest for those under 1 year of age and lower for those aged 5 to 9 years. (e) distribution of total prevalent cases; (f) distribution of total incident cases; (g) distribution of total death cases; (h) distribution of total DALYs. DALYs, disability-adjusted life years. (i–l): Correlations analysis of disease burden. The results showed that ASIR and ASDR in 1990 were negatively correlated with EAPC values during 1990-2021, whereas HDI values in 2021 were positively correlated with incidence EAPC values during 1990-2021. (i) correlation of ASIR (1990) and EAPCs; (j) correlation of ASDR (1990) and EAPCs; (k) correlation of HDI (2021) and incidence EAPC; (l) correlation of HDI (2021) and mortality EAPC (p > 0.05). ASPR, age-standardized prevalence rate; ASIR, age-standardized incidence rate; ASDR, age-standardized death rate; DALYs, Disability-Adjusted Life Years; HDI, human development index; EAPC, estimated annual percentage change; cor, correlation estimates. (m–p): Burden of hepatoblastoma for nations by Socio-demographic Index, 2021. In figures 1m and 1n, as SDI increased, ASPRs and ASIRs first showed a decreasing trend and then an increasing trend. In figures 1o and 1p, ASDRs and age-standardized DALYs rates showed a decreasing trend as SDI increases. (m) ASPR; (n) ASIR; (o) ASDR; (p) Age-standardized DALYs rate. ASPR, age-standardized prevalence rate; ASIR, age-standardized incidence rate; ASDR, age-standardized death rate; DALYs, Disability-Adjusted Life Years; SDI, Socio-demographic Index.

In 2021, there were 4048 (95% uncertainty interval [UI]: 3252 to 5000) new cases and 2416 (95% UI: 1922 to 3019) deaths globally. The ASPR, ASIR, ASDR, and age-standardized DALYs were 0.522 (95% UI: 0.279 to 0.685), 0.062 (95% UI: 0.05 to 0.077), 0.037 (95% UI: 0.029 to 0.046) and 3.274 (95% UI: 2.606 to 4.1) respectively, and the corresponding EAPC were −1.84% (95% confidence interval [CI]: −1.97 to −1.71), −2.03% (95% CI: −2.17 to −1.9), −2.54% (95% UI: −2.67 to −2.41) and −2.53% (95% CI: −2.66 to −2.4), respectively (Table 1). Fig. 1(a, b, c, and d) indicated the declining trends in ASPR, ASIR, ASDR and age-standardized DALYs rate by sex from 1990 to 2021. Fig. 1(e, f, g, and h) showed the global disease burden by age and gender in 2021. The prevalence, incidence, mortality cases, and DALYs were higher in the population under 1 year of age compared to other age groups, and females showed a heavier burden of disease in specific age subgroups.

**Table 1:** Global cases, age-standardized rates, estimated annual percentage change, average annual percentage change for hepatoblastoma.

Location	1990		2021		1990–2021	
	Estimated number (95% UI)	ASR per 100 000 (95% UI)	Estimated number (95% UI)	ASR per 100 000 (95% UI)	EAPC (95% CI)	AAPC(95% CI)
**Prevalence**						
Global	56 183 (20 484.3, 73 950.9)	0.907 (0.33, 1.196)	33 829 (18 007, 44 473)	0.522 (0.279, 0.685)	−1.84% (−1.97, − 1.71)	−1.76 (−1.88, − 1.63)
High SDI	2895.4 (2545.2, 3160)	0.47 (0.415, 0.512)	3114 (2799, 3428)	0.588 (0.529, 0.646)	0.82% (0.52, 1.12)	0.93 (0.71, 1.15)
High-middle SDI	8225.6 (4834.1, 10 305.1)	0.901 (0.531, 1.127)	3172 (2458, 4031)	0.475 (0.374, 0.604)	−2.53% (−2.87, − 2.19)	−2.02 (−2.66, − 1.39)
Middle SDI	21 126.8 (7491.7, 27 054.1)	1.061 (0.376, 1.358)	7001 (5042, 9324)	0.407 (0.294, 0.541)	−3.36% (−3.64, − 3.08)	−3.04 (−3.47, − 2.61)
Low-middle SDI	12 819.5 (3163.7, 18 818.9)	0.738 (0.182, 1.086)	9041 (3983, 11 915)	0.475 (0.21, 0.624)	−1.17% (−1.28, − 1.07)	−1.41 (−1.55, − 1.28)
Low SDI	11 091.7 (2435.8, 16 512.9)	1.205 (0.264, 1.812)	11 487 (2916, 16 898)	0.69 (0.175, 1.017)	−1.71% (−1.8, − 1.62)	−1.78 (−1.89, − 1.66)
High-income North America	991 (923.4, 1037.3)	0.456 (0.425, 0.478)	1621 (1419, 1820)	0.797 (0.699, 0.894)	2.13% (1.87, 2.38)	1.98 (1.62, 2.34)
Western Europe	947.2 (864.1, 1009.8)	0.414 (0.379, 0.441)	1087 (941, 1227)	0.519 (0.449, 0.587)	0.85% (0.67, 1.02)	0.84 (0.47, 1.22)
Australasia	55.9 (48.4, 63.2)	0.364 (0.315, 0.411)	119 (93, 152)	0.661 (0.513, 0.838)	2.05% (1.75, 2.35)	2.04 (0.81, 3.29)
Southern Latin America	28.6 (17.5, 36)	0.056 (0.034, 0.07)	32 (24, 42)	0.075 (0.057, 0.096)	1.6% (1.34, 1.87)	1.05 (0.59, 1.51)
**Incidence**						
Global	7063.7 (5798.8, 8280.7)	0.114 (0.094, 0.134)	4048(3252, 5000)	0.062 (0.05, 0.077)	−2.03%(−2.17, − 1.9)	−1.93 (−2.1, − 1.77)
High SDI	330.1 (312.2, 349.4)	0.053 (0.051, 0.057)	345(315, 374)	0.065 (0.059, 0.07)	0.73%(0.45, 1.02)	0.83 (0.62, 1.04)
High-middle SDI	996.9 (859.2, 1166.9)	0.109 (0.094, 0.128)	360(290, 448)	0.053 (0.043, 0.066)	−2.81%(−3.17, − 2.45)	−2.25 (−3.02, − 1.48)
Middle SDI	2649 (2302.6, 3055.9)	0.133 (0.116, 0.153)	823 (656, 1044)	0.047 (0.038, 0.06)	−3.62% (−3.91, − 3.33)	−3.28 (−3.64, − 2.91)
Low-middle SDI	1653.2 (1114.4, 2085.7)	0.095 (0.064, 0.12)	1102 (886, 1345)	0.058 (0.046, 0.071)	−1.38% (−1.48, − 1.29)	−1.61 (−1.77, − 1.46)
Low SDI	1431.5 (988.3, 1850.8)	0.156 (0.106, 0.204)	1417(996, 1917)	0.085 (0.06, 0.115)	−1.88% (−1.97, − 1.8)	−1.95 (−2.07, − 1.84)
High-income North America	110(107.1, 113)	0.1(0, 0.1)	179(160, 200)	0.1(0.1, 0.1)	2.1%(1.84, 2.35)	1.96(1.49, 2.43)
Western Europe	105.7(100.8, 110.8)	0.046(0.044, 0.048)	120(105, 136)	0.057(0.05, 0.065)	0.82%(0.65, 0.99)	0.8(0.43, 1.17)
Australasia	6.2(5.5, 6.9)	0.04(0.036, 0.045)	13(10, 17)	0.073(0.057, 0.092)	2.04%(1.75, 2.33)	2.02(0.78, 3.29)
Southern Latin America	3.5(3, 4)	0.007(0.006, 0.008)	4(3, 5)	0.009(0.007, 0.011)	1.47%(1.21, 1.73)	0.94(0.5, 1.38)
**Death**						
Global	4828.3(3938.6, 5670.5)	0.078(0.064, 0.092)	2416(1922, 3019)	0.037(0.029, 0.046)	−2.54%(−2.67, − 2.41)	−2.37(−2.48, − 2.26)
High SDI	144.4(133.1, 156.2)	0.023(0.021, 0.025)	85(78, 92)	0.016(0.014, 0.017)	−1.19%(−1.25, − 1.13)	−1.21(−1.29, − 1.13)
High-middle SDI	660.3(567.9, 774.4)	0.072(0.062, 0.085)	131(109, 159)	0.019(0.015, 0.023)	−5.13%(−5.56, − 4.7)	−4.15(−4.54, − 3.75)
Middle SDI	1842.2(1604.9, 2128.8)	0.092(0.081, 0.107)	459(369, 569)	0.026(0.021, 0.032)	−4.4%(−4.66, − 4.14)	−3.95(−4.26, − 3.64)
Low-middle SDI	1167(786.7, 1469.5)	0.067(0.045, 0.085)	752(604, 920)	0.039(0.032, 0.048)	−1.5%(−1.59, − 1.41)	−1.71(−1.82, − 1.59)
Low SDI	1012.4(700, 1308.5)	0.111(0.075, 0.144)	987(696, 1339)	0.059(0.042, 0.081)	−1.94%(−2.03, − 1.85)	−1.99(−2.11, − 1.88)
High-income North America	37.6(36.7, 38.5)	0.017(0.017, 0.018)	45(40, 50)	0.022(0.019, 0.024)	1.04%(0.91, 1.17)	1.14(1.02, 1.26)
Western Europe	40.9(39, 42.9)	0.018(0.017, 0.019)	27(24, 30)	0.013(0.011, 0.014)	−0.81%(−1.01, − 0.61)	−1.02(−1.27, − 0.76)
Australasia	2.1(1.8, 2.3)	0.013(0.012, 0.015)	3(2, 4)	0.017(0.013, 0.021)	1%(0.77, 1.22)	0.9(−0.09, 1.89)
Southern Latin America	2.3(2, 2.7)	0.005(0.004, 0.005)	2(2, 3)	0.005(0.004, 0.006)	0.69%(0.43, 0.94)	0.14(−0.28, 0.56)
**DALYs**						
Global	426 364.7(348 855.7, 500 155.9)	6.895(5.636, 8.091)	213 478(170 090, 267 250)	3.274(2.606, 4.1)	−2.53%(−2.66, − 2.4)	−2.37(−2.48, − 2.26)
High SDI	12 746.4(11 765.3, 13 782.2)	2.055(1.896, 2.223)	7604(6979, 8189)	1.405(1.288, 1.516)	−1.15%(−1.21, − 1.08)	−1.17(−1.25, − 1.09)
High-middle SDI	58 222(49 980, 68 442.9)	6.361(5.454, 7.479)	11 580(9597, 13 954)	1.664(1.373, 2.01)	−5.1%(−5.53, − 4.68)	−4.13(−4.52, − 3.74)
Middle SDI	162 544.9(141 567, 187 916.2)	8.159(7.105, 9.432)	40 299(32 391, 50 165)	2.29(1.842, 2.856)	−4.4%(−4.65, − 4.14)	−3.95(−4.27, − 3.64)
Low-middle SDI	102 973.2(69 477.7, 129 549.9)	5.944(3.995, 7.482)	66 393(53 247, 80 992)	3.475(2.784, 4.24)	−1.49%(−1.58, − 1.4)	−1.7(−1.81, − 1.59)
Low SDI	89 698.8(62 314.5, 115 953.6)	9.795(6.671, 12.756)	87 505(61 643, 118 846)	5.267(3.707, 7.159)	−1.93%(−2.02, − 1.84)	−1.99(−2.1, − 1.87)
High-income North America	3339.1(3259.6, 3417)	1.541(1.505, 1.577)	3992(3584, 4430)	1.932(1.732, 2.146)	1.07%(0.94, 1.2)	1.02(0.62, 1.41)
Western Europe	3629.8(3460.4, 3805.1)	1.575(1.501, 1.65)	2412(2144, 2711)	1.131(1.003, 1.274)	−0.77%(−0.97, − 0.58)	−0.97(−1.22, − 0.72)
Australasia	182.3(161.5, 202.8)	1.181(1.045, 1.314)	274(218, 339)	1.492(1.187, 1.839)	1.02%(0.8, 1.24)	0.92(−0.07, 1.93)
Southern Latin America	206.4(177.7, 238.3)	0.403(0.347, 0.465)	187(153, 225)	0.418(0.342, 0.505)	0.68%(0.42, 0.94)	0.13(−0.29, 0.55)

Data in cases and ASR represent the 95% uncertainty intervals, and data in EAPC and AAPC represent the 95% certain intervals. ASR, age-standardized rate; EAPC, estimated annual percentage change; APC, annual percent changes; AAPC, annual average percent changes; SDI, Socio-demographic Index.

## Regional level

Western Sub-Saharan Africa exhibited the heaviest disease burden in 2021, with an ASPR of 0.834 (95% UI: 0.247–1.21), an ASIR of 0.101 (95% UI: 0.073–0.135), an ASDR of 0.07 (95% UI: 0.051–0.093), and an age-standardized DALYs rate of 6.265 (95% UI: 4.526–8.292). However, despite these high values, a declining trend has been observed over the past 30 years. Unlike other regions, Australasia, High-income North America, Southern Latin America, and Western Europe exhibited an increasing trend in disease burden. Among these regions, High-income North America carried the heaviest burden (Table 1), the ASIR and ASDR were 0.088 (95% UI: 0.078 to 0.098) and 0.022 (95% UI: 0.019 to 0.024), with the EAPC of 2.1% (95% CI: 1.84 to 2.35) and 1.04% (95% CI: 0.91 to 1.17).

Among the five regions classified by SDI, the ASPR and ASIR in the high SDI region showed an increasing trend, with the EAPC of 0.82% (95% CI: 0.52 to 1.12) and 0.73% (95% CI: 0.45 to 1.02), while prevalence and incidence decreased in the other regions. In terms of mortality and DALYs, all five SDI regions exhibited a decrease (Table 1).

## National level

**Figure 2.**
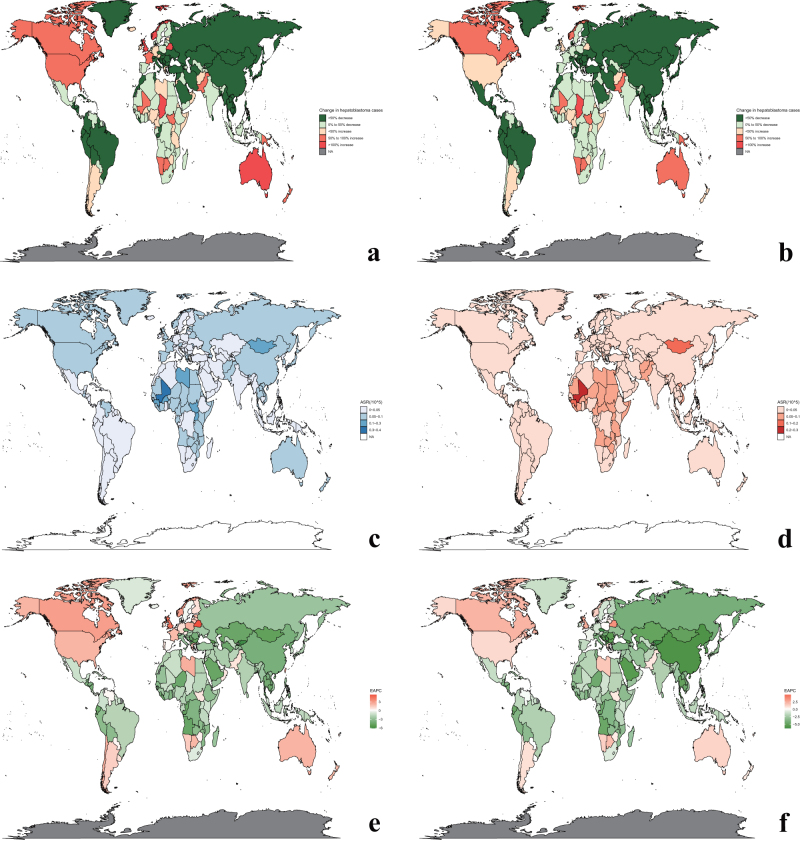
National level of hepatoblastoma burden. In (a) and (b), the number of incident cases and death cases of hepatoblastoma in 2021 at national level were shown compared to 1990. (c) and (d) showed the ASIRs and ASDRs of hepatoblastoma in 2021 at national level. (e) and (f) presented the incidence EAPCs and mortality EAPCs of hepatoblastoma over 1990–2021 at national level. (a) change in incident cases, 2021; (b) change in death cases, 2021; (c) ASIR, 2021; (d) ASDR, 2021; (e) incidence EAPC; (f) mortality EAPC. ASIR, age-standardized incidence rate; ASDR, age-standardized death rate; EAPC, estimated annual percentage change.

The highest ASIR in 2021 was observed in Mali (0.36; 95%UI: 0.209–0.539), followed by The Gambia (0.282; 95%UI: 0.183–0.42), Mongolia (0.268; 95%UI: 0.168–0.404), and Guinea (0.261; 95%UI: 0.148–0.403) (Fig. 2c). The countries with the highest EAPC of incidence were Belarus (5.38%; 95%CI: 4.6 to 6.16), followed by the United Kingdom (4.37%; 95%CI: 3.85 to 4.89), and Canada (2.76%; 95%CI: 2.4 to 3.13) (Fig. 2e). The highest ASDR in 2021 was observed in Mali (0.25; 95%UI: 0.146–0.376), followed by Gambi, Guinea, and Mongolia (Fig. 2d). The countries with the highest EAPC of death were Belarus (3.39%; 95%CI: 2.54 to 4.24), followed by the United Kingdom and Canada.

Consistent with the regional analysis (Supplementary Figure 2, http://links.lww.com/JS9/D845), we examined the relationship between age-standardized disease burden rates and SDI at the national level. We observed that as the SDI increases, prevalence and incidence initially decrease and subsequently increase. In contrast, mortality and DALYs rate exhibited a continuous declining trend (Fig. 1m, n, o, p).

Finally, correlation analyses indicated a negative correlation between the ASIR and ASDR in 1990 and their corresponding EAPCs, with higher initial rates associated with lower EAPCs (Fig. 1i, j). Conversely, there is a positive correlation between HDI and the EAPCs of incidence, with higher HDI values associated with higher incidence EAPC (Fig. 1k).

In summary, over the past 32 years, there has been a decline in the global burden of hepatoblastoma, contrasted with an annual increase in high SDI regions. GBD hepatoblastoma data was based on death cases under 10 years old, and the mortality rate for hepatoblastoma has decreased over the past 30 years due to advancements in chemotherapy and surgical techniques. This decline might explain the reduced burden of disease. Additionally, previous studies suggested that the main predisposing factors for hepatoblastoma include low birth weight, prematurity, specific genetic backgrounds, and maternal type 2 diabetes mellitus.^[^7-10^]^ These factors could account for the increased burden in high-SDI areas. However, there was a lack of global cohort studies on hepatoblastoma susceptibility factors, which should be the focus of future research.

## Data Availability

The data from this study can be accessed openly through the GBD 2021 online database.

## Supplementary Digital Content

Please see Fig. 1 (http://links.lww.com/JS9/D844); Fig. 3: http://links.lww.com/JS9/D846); Table 1 (http://links.lww.com/JS9/D847); Table 2 (http://links.lww.com/JS9/D848); Table 3 (http://links.lww.com/JS9/D849); Table 4 (http://links.lww.com/JS9/D850); Table 5 (http://links.lww.com/JS9/D851); Table 6 (http://links.lww.com/JS9/D852).
